# Triaqua­(cyclo­hex-4-ene-1,2-dicarboxyl­ato-κ*O* ^1^)(1,10-phenanthroline-κ^2^ *N*,*N*′)cobalt(II)

**DOI:** 10.1107/S160053681204024X

**Published:** 2012-10-20

**Authors:** Hai-Ye Li, Cheng Wang, Fu-Ping Huang, Yi-Min Jiang

**Affiliations:** a College of Chemistry and Chemical Engineering, Guangxi Normal University, Guilin, Guangxi 541004, People’s Republic of China

## Abstract

In the title compound, [Co(C_8_H_8_O_4_)(C_12_H_8_N_2_)(H_2_O)_3_], the Co^II^ atom is coordinated by two N atoms from a bidentate 1,10-phenanthroline ligand, one O atom from a monodentate 4-cyclo­hexene-1,2-dicarboxyl­ate ligand and three water O atoms in a distorted octa­hedral geometry. The mononuclear mol­ecules are engaged in extensive intra- and inter­molecular O—H⋯O hydrogen-bonding inter­actions and π–π stacking inter­actions [centroid–centroid distance = 3.784 (3) Å], forming a three-dimensional supra­molecular network.

## Related literature

For background to compounds with metal-organic framework structures, see: Huang *et al.* (2010 ▶); Ockwig *et al.* (2005 ▶); Rao *et al.* (2004 ▶). For a description of the Cambridge Structural Database (CSD), see: Allen (2002 ▶). For 4-cyclo­hexene-1,2-dicarboxyl­ates, see: Kim *et al.* (2004 ▶); Lee *et al.* (2006 ▶). For related structures, see: Baruah *et al.* (2007 ▶); Hou *et al.* (2007 ▶); Zhang *et al.* (2008 ▶).
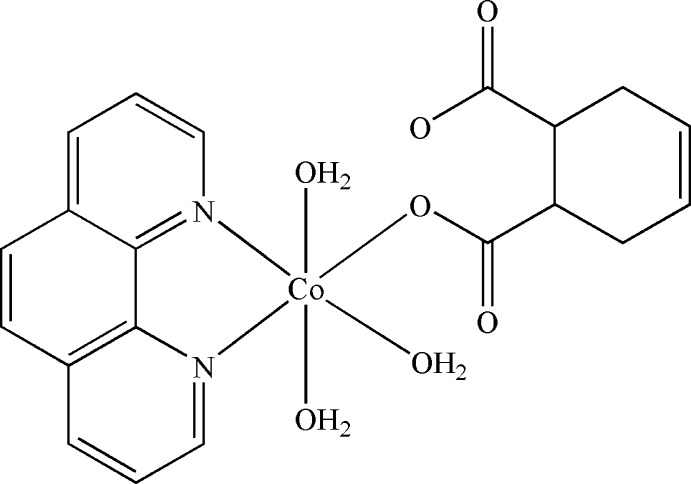


## Experimental

### 

#### Crystal data

[Co(C_8_H_8_O_4_)(C_12_H_8_N_2_)(H_2_O)_3_]
*M* *_r_* = 461.33
Monoclinic, 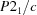

*a* = 8.1730 (16) Å
*b* = 20.210 (4) Å
*c* = 12.068 (2) Å
β = 91.44 (3)°
*V* = 1992.7 (7) Å^3^
*Z* = 4
Mo *K*α radiation
μ = 0.91 mm^−1^
*T* = 293 K
0.40 × 0.20 × 0.08 mm

#### Data collection

Bruker SMART CCD area-detector diffractometer
Absorption correction: multi-scan (*SADABS*; Sheldrick, 1996 ▶) *T*_min_ = 0.801, *T*_max_ = 0.945
15996 measured reflections
3606 independent reflections
2982 reflections with *I* > 2σ(*I*)
*R*_int_ = 0.073

#### Refinement

*R*[*F* ^2^ > 2σ(*F* ^2^)] = 0.061
*wR*(*F* ^2^) = 0.122
*S* = 0.99
3606 reflections
295 parameters
9 restraints
H atoms treated by a mixture of independent and constrained refinement
Δρ_max_ = 0.28 e Å^−3^
Δρ_min_ = −0.34 e Å^−3^

### 

Data collection: *SMART* (Bruker, 1998 ▶); cell refinement: *SAINT* (Bruker, 1998 ▶); data reduction: *SAINT*; program(s) used to solve structure: *SHELXS97* (Sheldrick, 2008 ▶); program(s) used to refine structure: *SHELXL97* (Sheldrick, 2008 ▶); molecular graphics: *SHELXTL* (Sheldrick, 2008 ▶); software used to prepare material for publication: *SHELXTL*.

## Supplementary Material

Crystal structure: contains datablock(s) global, I. DOI: 10.1107/S160053681204024X/zq2181sup1.cif

Structure factors: contains datablock(s) I. DOI: 10.1107/S160053681204024X/zq2181Isup2.hkl

Supplementary material file. DOI: 10.1107/S160053681204024X/zq2181Isup3.cdx

Additional supplementary materials:  crystallographic information; 3D view; checkCIF report

## Figures and Tables

**Table 1:** Hydrogen-bond geometry (Å, °)

*D*—H⋯*A*	*D*—H	H⋯*A*	*D*⋯*A*	*D*—H⋯*A*
O5—H5*A*⋯O3^i^	0.85 (3)	1.85 (3)	2.695 (4)	171 (4)
O5—H5*B*⋯O4	0.86 (4)	2.06 (4)	2.912 (4)	172 (4)
O6—H6*A*⋯O4^i^	0.86 (3)	1.86 (4)	2.716 (4)	174 (5)
O6—H6*B*⋯O3	0.86 (3)	1.99 (3)	2.835 (4)	165 (3)
O7—H7*A*⋯O2	0.86 (3)	1.77 (4)	2.610 (4)	165 (4)
O7—H7*B*⋯O4^ii^	0.86 (3)	1.87 (3)	2.734 (4)	175 (4)

Symmetry codes: (i) 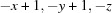; (ii) 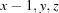.

**Fig. 1.**
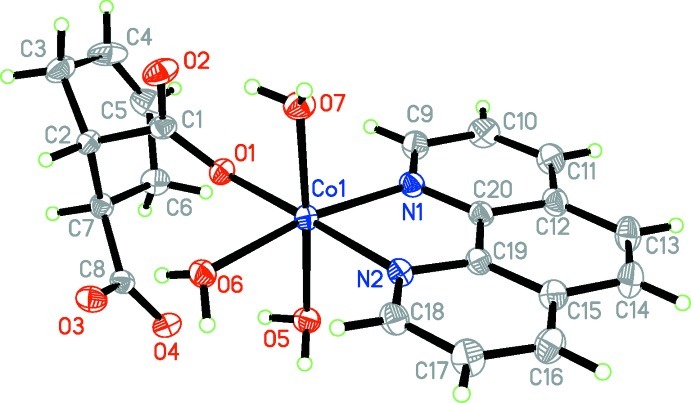
The asymmetric unit of the title compound with the atom-numbering scheme and displacement ellipsoids at the 30% probability level.

**Fig. 2.**
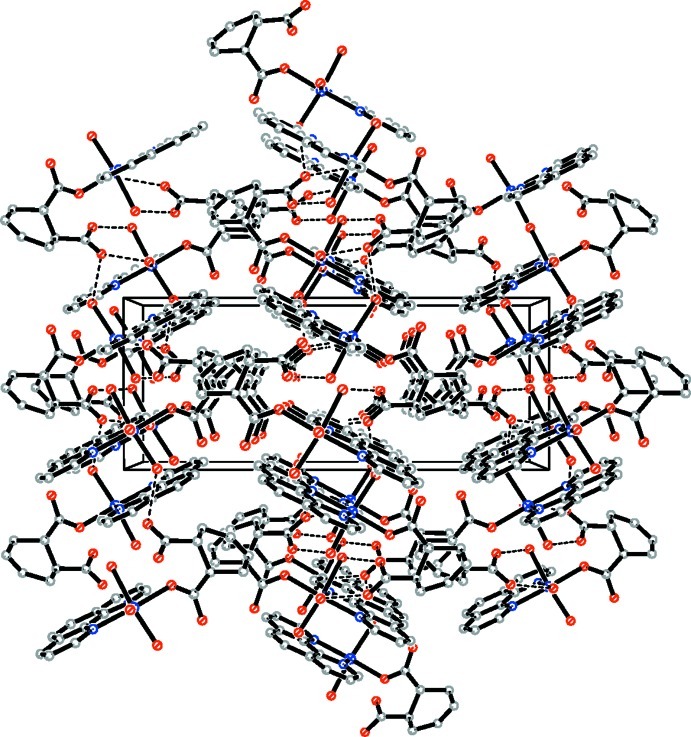
A packing diagram of the title compound. Hydrogen bonds are shown as dotted lines.

## supplementary crystallographic information

### Comment

Recently, carboxylic acid (as well as carboxylate) has been widely applied in engineering studies of inorganic-organic hybrid materials and the construction of metal-organic coordination supramolecular complexes (Huang *et al.*, 2010; Ockwig *et al.*, 2005; Rao *et al.*, 2004). Although the Cambridge Structural Database (Allen, 2002) contains a great number of transition metal derivatives of carboxylic acids, the metal derivatives of *cis*-4-cyclohexene-1,2-dicarboxylate acid documented are surprisingly rare (Kim *et al.*, 2004; Lee *et al.*, 2006). As part of this ongoing work, the title complex, [Co(C_8_H_8_O_4_)(C_12_H_8_N_2_)(H_2_O)_3_], has been prepared and structurally characterized (Fig. 1).

In the title compound, the Co^II^ atom is coordinated by two N atoms from a bidentate 1,10-phenanthroline ligand (*phen*), one O atom from a monodentate *cis*-4-cyclohexene-1,2-dicarboxylate ligand, and three O atoms from water molecules in a distorted octahedral geometry. The coordinating Co—N and Co—O bond lengths [Co—N 2.107 (3)–2.122 (3) Å; Co—O 2.071 (3)–2.152 (3) Å] agree well with those observed in analogous complexes (Baruah *et al.*, 2007; Hou *et al.*, 2007; Zhang *et al.*, 2008). The crystal packing (Fig. 2) exhibits intra- and inter-molecular O—H···O hydrogen bonds (Table 1) and *π–π* stacking interactions [Cg1···Cg2*^iii^* distance is 3.784 (3) Å (*iii* = -*x*, 1-*y*, 1-*z)* between the centroids of the (N1-C9-C10-C11-C12-C20) and (C12-C13-C14-C15-C19-C20) six-membered rings] forming a three-dimensional supramolecular network.

### Experimental

For the preparation of the title complex, *cis*-4-cyclohexene-1,2-dicarboxylate acid (0.085 g, 0.5 mmol), Co(NO_3_)_2_.6H_2_O (0.12 g, 0.5 mmol), *phen* (0.10 g, 0.5 mmol) and KHCO_3_ (0.10 g, 1 mmol) were dissolved in a water/ethanol solution (20 ml, 1:1). The solution was stirred for 3 h at room temperature and filtered. Red block-shaped crystals were obtained from the filtrate after 4 d.

### Refinement

H atoms of water molecules were located in a difference Fourier map and refined with distance restraints of O—H = 0.85 (2) Å and H···H = 1.39 (2) Å. All other H atoms were positoned geometrically and refined using a riding model, with C—H = 0.93 Å for C—H_aromatic_ and C—H = 0.97 Å for C—H_aliphatic_ [*U*_iso_(H) = 1.2*U*_eq_(C)] .

### Crystal data

**Table:**

[Co(C_8_H_8_O_4_)(C_12_H_8_N_2_)(H_2_O)_3_]	*F*(000) = 956
*M**_r_* = 461.33	*D*_x_ = 1.538 Mg m^−^^3^
Monoclinic, *P*2_1_/*c*	Mo *K*α radiation, λ = 0.71073 Å
Hall symbol: -P 2ybc	Cell parameters from 2567 reflections
*a* = 8.1730 (16) Å	θ = 1.5–25.3°
*b* = 20.210 (4) Å	µ = 0.91 mm^−^^1^
*c* = 12.068 (2) Å	*T* = 293 K
β = 91.44 (3)°	Block, red
*V* = 1992.7 (7) Å^3^	0.40 × 0.20 × 0.08 mm
*Z* = 4	

### Data collection

**Table:**

Bruker SMART CCD area-detector diffractometer	3606 independent reflections
Radiation source: fine-focus sealed tube	2982 reflections with *I* > 2σ(*I*)
Graphite monochromator	*R*_int_ = 0.073
phi and ω scans	θ_max_ = 25.3°, θ_min_ = 3.1°
Absorption correction: multi-scan (*SADABS*; Sheldrick, 1996)	*h* = −9→9
*T*_min_ = 0.801, *T*_max_ = 0.945	*k* = −24→24
15996 measured reflections	*l* = −14→14

### Refinement

**Table:**

Refinement on *F*^2^	Primary atom site location: structure-invariant direct methods
Least-squares matrix: full	Secondary atom site location: difference Fourier map
*R*[*F*^2^ > 2σ(*F*^2^)] = 0.061	Hydrogen site location: inferred from neighbouring sites
*wR*(*F*^2^) = 0.122	H atoms treated by a mixture of independent and constrained refinement
*S* = 0.99	*w* = 1/[σ^2^(*F*_o_^2^) + (0.0338*P*)^2^ + 5.*P*] where *P* = (*F*_o_^2^ + 2*F*_c_^2^)/3
3606 reflections	(Δ/σ)_max_ < 0.001
295 parameters	Δρ_max_ = 0.28 e Å^−^^3^
9 restraints	Δρ_min_ = −0.34 e Å^−^^3^

### Special details

**Table:**

Geometry. All e.s.d.'s (except the e.s.d. in the dihedral angle between two l.s. planes) are estimated using the full covariance matrix. The cell e.s.d.'s are taken into account individually in the estimation of e.s.d.'s in distances, angles and torsion angles; correlations between e.s.d.'s in cell parameters are only used when they are defined by crystal symmetry. An approximate (isotropic) treatment of cell e.s.d.'s is used for estimating e.s.d.'s involving l.s. planes.
Refinement. Refinement of *F*^2^ against ALL reflections. The weighted *R*-factor *wR* and goodness of fit *S* are based on *F*^2^, conventional *R*-factors *R* are based on *F*, with *F* set to zero for negative *F*^2^. The threshold expression of *F*^2^ > σ(*F*^2^) is used only for calculating *R*-factors(gt) *etc*. and is not relevant to the choice of reflections for refinement. *R*-factors based on *F*^2^ are statistically about twice as large as those based on *F*, and *R*- factors based on ALL data will be even larger.

### Fractional atomic coordinates and isotropic or equivalent isotropic displacement parameters (Å^2^)

**Table:**

	*x*	*y*	*z*	*U*_iso_*/*U*_eq_	
Co1	0.23393 (6)	0.53534 (3)	0.19466 (4)	0.02844 (17)	
C1	0.3182 (5)	0.67605 (19)	0.1389 (3)	0.0319 (9)	
C2	0.4497 (5)	0.71600 (19)	0.0814 (3)	0.0327 (9)	
H2A	0.4249	0.7143	0.0016	0.039*	
C3	0.4475 (6)	0.7890 (2)	0.1160 (4)	0.0463 (12)	
H3A	0.5051	0.8151	0.0620	0.056*	
H3B	0.3352	0.8045	0.1169	0.056*	
C4	0.5250 (6)	0.7988 (2)	0.2267 (5)	0.0567 (14)	
H4A	0.5006	0.8372	0.2655	0.068*	
C5	0.6267 (6)	0.7561 (3)	0.2732 (4)	0.0545 (14)	
H5C	0.6676	0.7657	0.3440	0.065*	
C6	0.6815 (5)	0.6932 (2)	0.2206 (4)	0.0400 (11)	
H6C	0.6419	0.6560	0.2632	0.048*	
H6D	0.8001	0.6916	0.2228	0.048*	
C7	0.6202 (5)	0.68620 (19)	0.1002 (3)	0.0319 (9)	
H7C	0.6947	0.7123	0.0553	0.038*	
C8	0.6262 (5)	0.61588 (19)	0.0556 (3)	0.0305 (9)	
C9	0.3023 (5)	0.5544 (2)	0.4478 (3)	0.0387 (10)	
H9A	0.3474	0.5952	0.4298	0.046*	
C10	0.3035 (6)	0.5346 (2)	0.5592 (4)	0.0460 (12)	
H10A	0.3491	0.5619	0.6138	0.055*	
C11	0.2385 (6)	0.4760 (2)	0.5869 (4)	0.0446 (12)	
H11A	0.2392	0.4628	0.6608	0.054*	
C12	0.1695 (5)	0.4345 (2)	0.5047 (3)	0.0369 (10)	
C13	0.0959 (6)	0.3723 (2)	0.5252 (4)	0.0446 (11)	
H13A	0.0915	0.3568	0.5976	0.054*	
C14	0.0322 (6)	0.3351 (2)	0.4419 (4)	0.0457 (12)	
H14A	−0.0140	0.2942	0.4577	0.055*	
C15	0.0348 (5)	0.3577 (2)	0.3300 (3)	0.0359 (10)	
C16	−0.0345 (5)	0.3215 (2)	0.2400 (4)	0.0426 (11)	
H16A	−0.0839	0.2807	0.2518	0.051*	
C17	−0.0280 (5)	0.3472 (2)	0.1365 (4)	0.0429 (11)	
H17A	−0.0732	0.3242	0.0764	0.051*	
C18	0.0467 (5)	0.4080 (2)	0.1210 (3)	0.0357 (10)	
H18A	0.0505	0.4246	0.0493	0.043*	
C19	0.1057 (5)	0.41830 (19)	0.3069 (3)	0.0295 (9)	
C20	0.1743 (5)	0.4581 (2)	0.3947 (3)	0.0306 (9)	
N1	0.2392 (4)	0.51715 (16)	0.3679 (3)	0.0312 (8)	
N2	0.1127 (4)	0.44351 (16)	0.2022 (3)	0.0295 (8)	
O1	0.3622 (3)	0.62360 (13)	0.1889 (2)	0.0315 (6)	
O2	0.1735 (3)	0.69566 (14)	0.1286 (3)	0.0439 (8)	
O3	0.5410 (3)	0.60345 (14)	−0.0292 (2)	0.0382 (7)	
O4	0.7164 (3)	0.57339 (13)	0.1038 (2)	0.0356 (7)	
O5	0.4681 (4)	0.48671 (15)	0.1938 (2)	0.0349 (7)	
O6	0.2453 (4)	0.53756 (15)	0.0198 (2)	0.0334 (7)	
O7	0.0207 (4)	0.59085 (15)	0.1985 (3)	0.0378 (7)	
H5A	0.465 (6)	0.4552 (16)	0.147 (3)	0.062 (17)*	
H6A	0.258 (5)	0.5009 (13)	−0.015 (4)	0.061 (17)*	
H7A	0.054 (5)	0.6283 (14)	0.174 (4)	0.052 (15)*	
H5B	0.538 (5)	0.5151 (17)	0.172 (4)	0.059 (17)*	
H6B	0.324 (4)	0.5630 (16)	0.000 (4)	0.054 (16)*	
H7B	−0.077 (3)	0.584 (2)	0.172 (4)	0.059 (16)*	

### Atomic displacement parameters (Å^2^)

**Table:**

	*U*^11^	*U*^22^	*U*^33^	*U*^12^	*U*^13^	*U*^23^
Co1	0.0293 (3)	0.0295 (3)	0.0264 (3)	−0.0004 (2)	0.0010 (2)	0.0015 (2)
C1	0.037 (3)	0.025 (2)	0.033 (2)	0.0006 (18)	0.0004 (18)	−0.0067 (18)
C2	0.039 (2)	0.028 (2)	0.031 (2)	0.0023 (18)	0.0014 (18)	0.0018 (18)
C3	0.044 (3)	0.027 (2)	0.069 (3)	0.001 (2)	0.008 (2)	0.004 (2)
C4	0.053 (3)	0.038 (3)	0.080 (4)	0.002 (2)	0.005 (3)	−0.028 (3)
C5	0.055 (3)	0.058 (3)	0.050 (3)	−0.009 (3)	−0.001 (2)	−0.030 (3)
C6	0.039 (3)	0.040 (3)	0.040 (3)	−0.005 (2)	−0.005 (2)	−0.008 (2)
C7	0.031 (2)	0.031 (2)	0.034 (2)	−0.0034 (18)	0.0025 (18)	0.0011 (18)
C8	0.028 (2)	0.030 (2)	0.034 (2)	−0.0042 (18)	0.0074 (18)	−0.0021 (18)
C9	0.045 (3)	0.038 (2)	0.033 (2)	−0.005 (2)	0.000 (2)	−0.0021 (19)
C10	0.059 (3)	0.047 (3)	0.032 (2)	−0.002 (2)	−0.010 (2)	−0.010 (2)
C11	0.057 (3)	0.050 (3)	0.027 (2)	0.002 (2)	−0.003 (2)	0.001 (2)
C12	0.039 (3)	0.042 (3)	0.030 (2)	0.002 (2)	0.0023 (19)	0.0049 (19)
C13	0.054 (3)	0.050 (3)	0.030 (2)	−0.004 (2)	−0.001 (2)	0.011 (2)
C14	0.054 (3)	0.043 (3)	0.040 (3)	−0.009 (2)	0.002 (2)	0.011 (2)
C15	0.035 (2)	0.038 (2)	0.035 (2)	−0.0007 (19)	−0.0006 (19)	0.0013 (19)
C16	0.040 (3)	0.037 (3)	0.051 (3)	−0.011 (2)	−0.001 (2)	0.002 (2)
C17	0.043 (3)	0.049 (3)	0.036 (3)	−0.012 (2)	−0.005 (2)	−0.004 (2)
C18	0.038 (2)	0.043 (3)	0.026 (2)	−0.003 (2)	−0.0022 (18)	−0.0007 (19)
C19	0.027 (2)	0.033 (2)	0.028 (2)	−0.0013 (17)	0.0009 (17)	0.0007 (17)
C20	0.030 (2)	0.035 (2)	0.026 (2)	0.0045 (18)	0.0017 (17)	0.0014 (18)
N1	0.033 (2)	0.0333 (19)	0.0272 (19)	0.0009 (15)	0.0018 (15)	−0.0008 (15)
N2	0.0291 (18)	0.0335 (19)	0.0261 (18)	−0.0026 (15)	0.0024 (14)	−0.0007 (14)
O1	0.0310 (15)	0.0284 (15)	0.0349 (16)	−0.0012 (12)	−0.0008 (12)	0.0018 (12)
O2	0.0292 (17)	0.0342 (17)	0.068 (2)	0.0063 (13)	−0.0034 (15)	0.0008 (15)
O3	0.0409 (17)	0.0386 (17)	0.0349 (17)	0.0004 (13)	−0.0023 (14)	−0.0103 (13)
O4	0.0337 (16)	0.0298 (16)	0.0432 (18)	0.0029 (13)	−0.0031 (13)	−0.0023 (13)
O5	0.0360 (18)	0.0356 (17)	0.0329 (17)	0.0033 (14)	0.0010 (13)	−0.0023 (14)
O6	0.0376 (18)	0.0334 (17)	0.0293 (16)	−0.0010 (14)	0.0023 (12)	−0.0015 (14)
O7	0.0280 (17)	0.0381 (19)	0.0474 (19)	0.0029 (14)	0.0000 (14)	0.0003 (15)

### Geometric parameters (Å, º)

**Table:**

Co1—O1	2.071 (3)	C9—H9A	0.9300
Co1—O7	2.074 (3)	C10—C11	1.345 (6)
Co1—N2	2.107 (3)	C10—H10A	0.9300
Co1—O6	2.115 (3)	C11—C12	1.407 (6)
Co1—N1	2.122 (3)	C11—H11A	0.9300
Co1—O5	2.152 (3)	C12—C20	1.412 (5)
C1—O2	1.250 (5)	C12—C13	1.418 (6)
C1—O1	1.268 (5)	C13—C14	1.348 (6)
C1—C2	1.525 (6)	C13—H13A	0.9300
C2—C7	1.530 (6)	C14—C15	1.427 (6)
C2—C3	1.533 (6)	C14—H14A	0.9300
C2—H2A	0.9800	C15—C19	1.387 (6)
C3—C4	1.477 (7)	C15—C16	1.415 (6)
C3—H3A	0.9700	C16—C17	1.355 (6)
C3—H3B	0.9700	C16—H16A	0.9300
C4—C5	1.315 (7)	C17—C18	1.386 (6)
C4—H4A	0.9300	C17—H17A	0.9300
C5—C6	1.494 (6)	C18—N2	1.319 (5)
C5—H5C	0.9300	C18—H18A	0.9300
C6—C7	1.531 (6)	C19—N2	1.364 (5)
C6—H6C	0.9700	C19—C20	1.433 (5)
C6—H6D	0.9700	C20—N1	1.349 (5)
C7—C8	1.521 (5)	O5—H5A	0.852 (19)
C7—H7C	0.9800	O5—H5B	0.854 (19)
C8—O3	1.250 (5)	O6—H6A	0.860 (19)
C8—O4	1.264 (5)	O6—H6B	0.859 (19)
C9—N1	1.319 (5)	O7—H7A	0.859 (18)
C9—C10	1.403 (6)	O7—H7B	0.858 (19)
			
O1—Co1—O7	87.78 (12)	N1—C9—H9A	119.0
O1—Co1—N2	177.61 (12)	C10—C9—H9A	119.0
O7—Co1—N2	94.53 (13)	C11—C10—C9	119.8 (4)
O1—Co1—O6	85.03 (11)	C11—C10—H10A	120.1
O7—Co1—O6	93.97 (12)	C9—C10—H10A	120.1
N2—Co1—O6	95.43 (12)	C10—C11—C12	120.3 (4)
O1—Co1—N1	100.63 (12)	C10—C11—H11A	119.9
O7—Co1—N1	93.88 (12)	C12—C11—H11A	119.9
N2—Co1—N1	78.61 (12)	C11—C12—C20	116.2 (4)
O6—Co1—N1	170.49 (12)	C11—C12—C13	124.7 (4)
O1—Co1—O5	86.69 (11)	C20—C12—C13	119.0 (4)
O7—Co1—O5	174.34 (13)	C14—C13—C12	121.4 (4)
N2—Co1—O5	90.99 (12)	C14—C13—H13A	119.3
O6—Co1—O5	86.76 (11)	C12—C13—H13A	119.3
N1—Co1—O5	85.96 (12)	C13—C14—C15	120.9 (4)
O2—C1—O1	124.7 (4)	C13—C14—H14A	119.6
O2—C1—C2	117.6 (4)	C15—C14—H14A	119.6
O1—C1—C2	117.7 (4)	C19—C15—C16	117.6 (4)
C1—C2—C7	112.0 (3)	C19—C15—C14	119.3 (4)
C1—C2—C3	111.9 (3)	C16—C15—C14	123.1 (4)
C7—C2—C3	110.8 (3)	C17—C16—C15	119.0 (4)
C1—C2—H2A	107.3	C17—C16—H16A	120.5
C7—C2—H2A	107.3	C15—C16—H16A	120.5
C3—C2—H2A	107.3	C16—C17—C18	119.4 (4)
C4—C3—C2	111.6 (4)	C16—C17—H17A	120.3
C4—C3—H3A	109.3	C18—C17—H17A	120.3
C2—C3—H3A	109.3	N2—C18—C17	123.8 (4)
C4—C3—H3B	109.3	N2—C18—H18A	118.1
C2—C3—H3B	109.3	C17—C18—H18A	118.1
H3A—C3—H3B	108.0	N2—C19—C15	122.9 (4)
C5—C4—C3	123.3 (4)	N2—C19—C20	116.8 (3)
C5—C4—H4A	118.3	C15—C19—C20	120.3 (4)
C3—C4—H4A	118.3	N1—C20—C12	123.0 (4)
C4—C5—C6	124.8 (5)	N1—C20—C19	117.9 (3)
C4—C5—H5C	117.6	C12—C20—C19	119.1 (4)
C6—C5—H5C	117.6	C9—N1—C20	118.6 (3)
C5—C6—C7	112.8 (4)	C9—N1—Co1	128.3 (3)
C5—C6—H6C	109.0	C20—N1—Co1	113.0 (3)
C7—C6—H6C	109.0	C18—N2—C19	117.3 (3)
C5—C6—H6D	109.0	C18—N2—Co1	129.1 (3)
C7—C6—H6D	109.0	C19—N2—Co1	113.6 (2)
H6C—C6—H6D	107.8	C1—O1—Co1	126.8 (2)
C8—C7—C2	110.7 (3)	Co1—O5—H5A	109 (3)
C8—C7—C6	114.2 (3)	Co1—O5—H5B	107 (3)
C2—C7—C6	112.3 (3)	H5A—O5—H5B	108 (3)
C8—C7—H7C	106.4	Co1—O6—H6A	119 (3)
C2—C7—H7C	106.4	Co1—O6—H6B	110 (3)
C6—C7—H7C	106.4	H6A—O6—H6B	107 (3)
O3—C8—O4	123.2 (4)	Co1—O7—H7A	101 (3)
O3—C8—C7	117.1 (4)	Co1—O7—H7B	133 (3)
O4—C8—C7	119.7 (4)	H7A—O7—H7B	108 (3)
N1—C9—C10	122.1 (4)		
			
O2—C1—C2—C7	−179.6 (3)	N2—C19—C20—N1	0.4 (5)
O1—C1—C2—C7	−3.0 (5)	C15—C19—C20—N1	−178.9 (4)
O2—C1—C2—C3	55.3 (5)	N2—C19—C20—C12	179.7 (3)
O1—C1—C2—C3	−128.1 (4)	C15—C19—C20—C12	0.4 (6)
C1—C2—C3—C4	77.6 (5)	C10—C9—N1—C20	−0.1 (6)
C7—C2—C3—C4	−48.1 (5)	C10—C9—N1—Co1	−177.0 (3)
C2—C3—C4—C5	20.1 (7)	C12—C20—N1—C9	0.5 (6)
C3—C4—C5—C6	1.7 (9)	C19—C20—N1—C9	179.7 (4)
C4—C5—C6—C7	5.8 (7)	C12—C20—N1—Co1	177.9 (3)
C1—C2—C7—C8	59.8 (4)	C19—C20—N1—Co1	−2.9 (4)
C3—C2—C7—C8	−174.5 (3)	O1—Co1—N1—C9	2.4 (4)
C1—C2—C7—C6	−69.1 (4)	O7—Co1—N1—C9	−86.0 (4)
C3—C2—C7—C6	56.6 (5)	N2—Co1—N1—C9	−179.9 (4)
C5—C6—C7—C8	−161.8 (4)	O5—Co1—N1—C9	88.3 (4)
C5—C6—C7—C2	−34.8 (5)	O1—Co1—N1—C20	−174.6 (3)
C2—C7—C8—O3	34.0 (5)	O7—Co1—N1—C20	96.9 (3)
C6—C7—C8—O3	161.9 (4)	N2—Co1—N1—C20	3.1 (3)
C2—C7—C8—O4	−146.7 (4)	O5—Co1—N1—C20	−88.7 (3)
C6—C7—C8—O4	−18.9 (5)	C17—C18—N2—C19	−0.1 (6)
N1—C9—C10—C11	−0.2 (7)	C17—C18—N2—Co1	178.9 (3)
C9—C10—C11—C12	0.1 (7)	C15—C19—N2—C18	0.7 (6)
C10—C11—C12—C20	0.3 (7)	C20—C19—N2—C18	−178.6 (4)
C10—C11—C12—C13	−179.0 (5)	C15—C19—N2—Co1	−178.4 (3)
C11—C12—C13—C14	−180.0 (5)	C20—C19—N2—Co1	2.3 (4)
C20—C12—C13—C14	0.7 (7)	O7—Co1—N2—C18	85.1 (4)
C12—C13—C14—C15	−0.8 (7)	O6—Co1—N2—C18	−9.4 (4)
C13—C14—C15—C19	0.7 (7)	N1—Co1—N2—C18	178.1 (4)
C13—C14—C15—C16	−178.1 (4)	O5—Co1—N2—C18	−96.2 (4)
C19—C15—C16—C17	0.4 (6)	O1—Co1—N2—C19	69 (3)
C14—C15—C16—C17	179.2 (4)	O7—Co1—N2—C19	−95.9 (3)
C15—C16—C17—C18	0.2 (7)	O6—Co1—N2—C19	169.6 (3)
C16—C17—C18—N2	−0.4 (7)	N1—Co1—N2—C19	−2.9 (3)
C16—C15—C19—N2	−0.9 (6)	O5—Co1—N2—C19	82.8 (3)
C14—C15—C19—N2	−179.8 (4)	O2—C1—O1—Co1	34.6 (6)
C16—C15—C19—C20	178.4 (4)	C2—C1—O1—Co1	−141.7 (3)
C14—C15—C19—C20	−0.5 (6)	O7—Co1—O1—C1	−36.1 (3)
C11—C12—C20—N1	−0.6 (6)	O6—Co1—O1—C1	58.1 (3)
C13—C12—C20—N1	178.8 (4)	N1—Co1—O1—C1	−129.6 (3)
C11—C12—C20—C19	−179.8 (4)	O5—Co1—O1—C1	145.1 (3)
C13—C12—C20—C19	−0.4 (6)		

### Hydrogen-bond geometry (Å, º)

**Table:**

*D*—H···*A*	*D*—H	H···*A*	*D*···*A*	*D*—H···*A*
O5—H5*A*···O3^i^	0.85 (3)	1.85 (3)	2.695 (4)	171 (4)
O5—H5*B*···O4	0.86 (4)	2.06 (4)	2.912 (4)	172 (4)
O6—H6*A*···O4^i^	0.86 (3)	1.86 (4)	2.716 (4)	174 (5)
O6—H6*B*···O3	0.86 (3)	1.99 (3)	2.835 (4)	165 (3)
O7—H7*A*···O2	0.86 (3)	1.77 (4)	2.610 (4)	165 (4)
O7—H7*B*···O4^ii^	0.86 (3)	1.87 (3)	2.734 (4)	175 (4)

Symmetry codes: (i) −*x*+1, −*y*+1, −*z*; (ii) *x*−1, *y*, *z*.
